# Signifying the Relationship between Fear of COVID-19, Psychological Concerns, Financial Concerns and Healthcare Employees Job Performance: A Mediated Model

**DOI:** 10.3390/ijerph19052657

**Published:** 2022-02-24

**Authors:** Muddassar Sarfraz, Xiangbo Ji, Muhammad Asghar, Larisa Ivascu, Ilknur Ozturk

**Affiliations:** 1 College of International Students, Wuxi University, Wuxi 214105, China; muddassar.sarfraz@gmail.com (M.S.); jixiangbo010@163.com (X.J.); 2 School of Economics and Management, Yanshan University, Qinhuangdao 066104, China; asghargcufpk@gmail.com; 3 Department of Management, Faculty of Management in Production and Transportation, Politehnica University of Timisoara, 300191 Timisoara, Romania; 4 Higher Vocational School, Cag University, Mersin 33800, Turkey; ilknurozturk@cag.edu.tr

**Keywords:** fear of COVID-19, depression, anxiety, stress, job performance, healthcare

## Abstract

The coronavirus pandemic (COVID-19) has undoubtedly created immense health problems in the global healthcare sector. Apart from its impact on physical health, it has devastatingly affected the psychological well-being of individuals. Based on Affective Events Theory (AET), the current study aims to contemplate the relationship between Fear of COVID-19 (CVF), psychological concerns (PC), and financial concerns (FC) while considering the impact on the healthcare employees’ job performance (JP). Moreover, this study investigates the mediating role of job anxiety (ANX), stress (ST), and depression (DEP). The data were collected through an online structured questionnaire (Google Forms) from 489 employees working in the healthcare centers of Pakistan. The structured equation modeling (partial least square) approach is adopted for data analysis. The study results showed that Fear of COVID-19, psychological and financial concerns positively and significantly affect healthcare workers’ job performance. Depression, anxiety, and stress mediated the relationship between Fear of COVID-19, psychological concerns, financial concerns, and job performance. The study theoretically and practically contributes to the existing literature on psychological and mental health by providing a better understanding of the individual variables that affect employees’ job performance.

## 1. Introduction

In December 2019, the severe acute respiratory syndrome coronavirus (SARS-CoV-2) emerged from China, affecting every domain of human lives [1]. The profound representation of the hideous infection has made the world’s healthcare division face psychological vulnerability. The COVID-19 high efficacy disrupted human settlement, accelerating the world’s mortality rate causing an extreme toll on the healthcare industry. The prolonged continuation of the malignant infection massively exasperated global health repercussions, thus illuminating this event as a universal health emergency by the World Health Organization [2].

Verily, COVID-19 is a mystery that provoked the entire nation to experience its devastating effects on individuals’ psychological health. Accordingly, pandemic fear has emerged as the most disrupting factor interrupting the employees’ work role across worldwide organizations [3]. Recently, this pathogen brought the world’s biggest psychological problem affecting universal healthcare performance [4]. Moreover, the high COVID-19 infectivity (i.e., fear) has significantly made individuals worry about its health repercussions, thus deteriorating psychological well-being [5].

In particular, psychological well-being refers to the state of wellness that makes the individuals benefit from their abilities, thus dealing with life stresses (e.g., depression, anxiety, and stress) [6]. The COVID-19 pandemic has overwhelmed the health sector beyond the normal functioning to sensitive caretaking. This progressing widespread appeared to bring devastating effects on global economies, with Pakistan facing substantial setbacks [7]. In particular, the mental toll of COVID-19 challenged the frontline workers to deal with high-stress situations such as long working hours, thus influencing normal healthcare functioning [8].

Poor mental health conditions adversely affect the individual’s workplace activities. Accordingly, the COVID-19 psychological concern shows that increased depression, anxiety, and stress unduly record varying degrees of vulnerability among people [9]. In support, the study shows that the healthcare workforce feels the danger of becoming infected, subsequently losing confidence in the global healthcare system to prevent the disease [10]. This inadequacy in treating the virus promoted severe health issues (e.g., depression, anxiety, and stress) among health professionals [11]. The COVID-19 isolation has made the workforce disconnect from family and friends, subsequently experiencing helplessness, anxiety, and distress [12]. Consistently, the study conducted in Pakistan by Imran [13] showed that healthcare employees during the COVID-19 pandemic immensely experienced the feeling of health disorders, including depression (26.4%), anxiety (22.6%), and stress (4.4%), respectively [13].

Therefore, in addition to worrying about psychological concerns, the COVID-19 fast transmission, its increasing infectivity, and psychological distress elevated the feeling of financial depression among employees [14]. The COVID-19 financial hit affected various communities, with its extending effect on millions of individuals who were traumatized due to massive layoffs [15]. The colossal turbulence of the pandemic has made the healthcare workers lose jobs, thus promoting the feeling of financial anxiety among the individuals [3]. This critical situation led the employees to face economic vulnerability, leading to a sense of helplessness and grief. Verily, the unexpected consequences of the pandemic led the hospitality industry to experience global economic depression, engendering an increase in the level of job insecurity [16]. The unstable economic composition of COVID-19 created financial distress among individuals, making 21.1% lose their jobs [17]. Perhaps, to deal with the current economic repression, an effective mechanism needs to be adopted for ensuring an immediate response to the emerging concerns.

Considerably, after reviewing the literature on the COVID-19 pandemic, it is clear that immense research is available, but, in the context of possible predictors of psychological and financial concerns, research needs to be performed on the vulnerable population (i.e., the healthcare workforce) [18]. Several studies explored the impact of the COVID-19 pandemic. Based on Affective Events Theory (AET), this study is the pioneer, assessing the multiple factors such as the level of COVID-19 fear, psychological concerns, and financial concerns affecting the work performance of the frontline workers. Indeed, COVID-19 is an infectious disease damaging the workers’ welfare with little attention to psychological and financial concerns. Affective events theory is based on the idea that people are emotional beings and influence their behavior. Workplace stress, emotions, and sentiments are linked to job performance through the Affective Events Theory (AET) [19]. According to Martocchio [20], the affective events theory (AET) posits that emotions are critically important to handling workplace situations. According to the theory, employee performance, engagement, and satisfaction are all impacted by the underlying factors and behaviors (such as feelings) they experience during their work. However, in this challenging phase, this study holds great significance in exploring the various factors influencing the employees’ performance in the healthcare industry.

Fundamentally, the purpose of the paper is to provide an evidence-based view of the adverse effect of mental health on frontline health employees during the time of pandemic crises. This article sheds light on the challenging working conditions and the importance of safeguarding the employees’ psychological wellness. This article addresses the Fear of COVID-19 sustainability, specifically recording psychological and economic concerns. Healthcare workers bear a heavy mental and financial crisis, directly affecting their job performance. Perhaps, to support psychological well-being, this study aims to explore the role of psychological factors affecting the mental health of healthcare employees, thus influencing their work performance during the pandemic period. Furthermore, to test the potential determinants of the psychological problems, the study analyzes the mediating effect of depression, anxiety, and stress on individuals’ work performance. Altogether, the study goal is to assess the fear level of health workers concerning the widespread coronavirus and establish scientific literature on the psychological and financial concerns associated with individual job performance.

Affective events theory is based on the idea that internal changes such as sentiments and thoughts influence employee happiness, organizational commitment, and work performance. According to the AET, employees’ sentiments and emotions characterize the working environment, despite cognitive-based behaviors being the best signs of job success [21]. Based on AET, it is possible to recognize and affect the mental performance of employees at work by identifying both positive and bad events. In other words, this refers to the impacts and outcomes of internal cognition (such as sentiments, perceptions, and psychological states), as well as levels of dedication, contentment in one’s work, and productivity [22].

The study will follow the next structure. The first section provides a brief overview of the topic. Section 2 (i.e., Literature review) proposes the theoretical framework for hypothesis development and testing. Section 3 (Methodology) suggests the appropriate analysis and statistical procedure, while Section 4 explains the study findings. In the same vein, Section 5 and Section 6 illustrate the research discussion and conclusion, respectively.

## 2. Theoretical Background and Hypothesis Development

### 2.1. Concept and Consequences of COVID-19 Fear

Fear is an adaptive human feeling that fights against potential danger [23]. The unsavory feeling brings detrimental effects on the individuals’ psychological health. Fear harms the employees’ mental well-being, subsequently elevating the psychiatric symptoms of depression. Depression is a disorder that aggravates the symptoms of low morale, grief, sadness, and distress, adversely affecting the individual’s mental health [24].

Over the years, the increased long-term epidemics have aroused the feeling of depression in the population. Today, the coronavirus accelerated the fear in individuals [25], thereby increasing the responsiveness of depression among the employees [26]. In particular, during the pandemic, the healthcare workers have become extremely exhausted due to prolonged working hours. The increasing COVID-19 significantly exacerbated the symptoms of depression among the individuals, unfavorably affecting the employees’ psychological health. In support, the research exploring the psychological symptoms associated with the pandemic reports demonstrates a high prevalence of depression (28%) and anxiety (33%) in the healthcare workers in China [27].

In contrast, the Fear of COVID-19 substantially influenced the employee outcome. Given the articulation, the study shows that increased COVID-19 worry led the health workforce to exhibit poor work performance, thus drastically altering the workplace dynamics [28]. The vast fear of the COVID-19 infectivity manifested the frontline representatives to bear excessive workload, thus hampering their job performance. The heightened Fear of COVID-19 recorded a decline in workers’ performance due to the increasing psychological problems [29]. In particular, a healthy mental state influences the employees’ work outcomes. The current pandemic has posed significant occupational challenges for healthcare staff, making them perform their duties day and night. Indeed, the pandemic exacerbated the psychological issues, causing healthcare workers difficulty performing job tasks [30].

Fear demands a defensive response [23]. When the fear is uncontrollable, it upgrades into the feeling of anxiety. In recent years, the increased COVID-19 fear has taken an intense emotional toll on employees’ psychological health, essentially making the individuals work with an anxiety disorder [31]. The COVID-19 fear caused severe health challenges, thus affecting the lives of the caretakers. In support, Mertens’ [32] study reveals that the terrifying characteristics of the pandemic triggered the element of anxiety and fear in the health workers [32]. Prolonged working hours during the pandemic increased the work intensity of the healthcare staff (i.e., nurses). Accordingly, Imran’s [13] study shows that health professionals working in Pakistan during the COVID-19 pandemic have encountered extreme anxiety (7.2%) and distress, thus adversely affecting their mental health.

Furthermore, the 2019 coronavirus declared the pandemic as the significant element boosting the stress symptoms in individuals. Stress is a defensive response that requires physical, emotional, and psychological adaptation [33]. Individuals respond to stress differently based on emotional, physical, and psychological factors. The COVID-19 adversity (i.e., fear) drastically influenced individuals’ psychological health, causing them to face significant distress. The increased emotional exhaustion, loss of energy, and fatigue negatively affected individuals [34], thus increasing their inability to cope with the COVID-19 stressors. In particular, the COVID-19 fear immensely exposed the health workers to vulnerable effects of psychological stress [35]. The COVID-19 situation led healthcare employees to react to stressful situations, thus detrimentally influencing their psychological well-being. In the illustration, the study shows that 112 million individuals reported the symptoms of extreme stress in China [36]. There are both good and negative events when it comes to affecting an employee’s mood and job satisfaction. AET is only concerned with the impact that the workplace has on the emotional health of its employees, and it makes no allowances for the influence of outside circumstances. It is not included by the AET definition if an employee’s child falls sick at home because it occurs outside the workplace [21,22]. The high stress during the pandemic significantly reports an increased mortality rate, poor health outcomes, and lower quality of life. Therefore, it is critical to understand the relationship between the psychological problems (e.g., depression, anxiety, stress) to improve the employees’ health and job performance. Consequently, based on the previous literature, the hypotheses developed are as follows:

**Hypothesis** **1** **(1a):** *COVID-19 Fear has a positive and significant impact on depression.*

**Hypothesis** **1** **(1b):** *COVID-19 Fear has a negative and significant impact on job performance*.

**Hypothesis** **1** **(1c):** *COVID-19 Fear has a positive and significant impact on anxiety*.

**Hypothesis** **1** **(1d):** *COVID-19 Fear has a positive and significant impact on stress*.

### 2.2. Psychological Concerns

During the pandemic, the increasing psychological concern accelerated the symptoms of depression among individuals. The expanding health concerns profoundly enhanced the relationship between the COVID-19 outbreak and depression. The rising psychological worries fostered the relationship between employees’ well-being and depression. The negative experience of employees’ psychological health (i.e., fatigue, emotional exhaustion) made the healthcare employees fear for their psychological wellness. Accordingly, the study indicates that people with high depression exhibit feelings of emotional exhaustion, fatigue, and energy loss [37]. The psychological concerns widely report workplace detachment, thus cultivating distress among the healthcare workers [38]. Indeed, the recent pandemic caused health professionals to endure the powerful effect of the COVID-19 psychological pressure. It frightened the frontline workers about their psychological well-being and social life [39]. Therefore, psychological concerns need to be emphasized, thus coping with the challenging demands of the workplace.

Today, ensuring the employees’ psychological well-being has become the prime area of concern for many researchers. Acknowledging the focal point of safeguarding the workers’ well-being is critically needed to ensure rapid recovery from the COVID-19 situation. Satisfying the psychological concerns makes the individual meet the work demands, thus regaining the lost energy. In particular, the research shows that mental health concerns lower the individual work performance and increase the chances of more mistakes. Such conditions negatively influence task performance, thus promoting the symptoms of distress and illness among the individual [40]. Considerably, during the COVID-19 outbreak, the psychological strain (i.e., depression) led to declines in the employees’ health conditions [41]. The increased psychological concerns during the pandemic made employees encounter excessive workload and pressure [42], thus affecting their job performance.

Considerably, in the COVID-19 era, the frontline healthcare representatives have become vulnerable to psychological issues such as anxiety. Given the illustration, the recent research involving 97,333 workers from 21 different countries records a high prevalence of anxiety symptoms (22.1%) among healthcare workers [43]. During COVID-19, the possibility of making the family and friends infected traumatized the workers [10]. Verily, workplace wellness is vital for successfully delivering safe health services. Positive psychological well-being gained significant consideration from health scholars, recognizing the need for the employees’ wellness. Perhaps, beyond the feeling of good to functioning well, psychological well-being contributes towards employee happiness, pleasures, and personal growth [44].

Accordingly, evidence linked to psychological stress was recorded during the COVID-19 period. In the illustration, the study showed that the uncertain situation of the pandemic made the workers lose control over their lives and work [45]. The wide spread of the disease made the workers stressed about its extending infectivity [46]. The COVID-19 pandemic brought significant psychological concerns affecting the wellness of individuals. Given the articulation, the study shows that during the COVID-19 outbreak, a dominant proportion of the health workforce experienced symptoms of stress, subsequently harming their psychological well-being [47]. Hence, based on the prior studies, the literature concludes the following hypotheses.

**Hypothesis** **2** **(2a):** *Psychological concerns have a positive and significant impact on depression*.

**Hypothesis** **2** **(2b):** *Psychological concerns have a negative and significant impact on job performance*.

**Hypothesis** **2** **(2c):** *Psychological concerns have a positive and significant impact on anxiety*.

**Hypothesis** **2** **(2d):** *Psychological concerns have a positive and significant impact on stress*.

### 2.3. Financial Concerns

The financial burden has a profound effect on an individual’s psychological health. Depression is a predominant mental disorder that makes the individual lose interest in daily activities, inflaming the feeling of sadness among the employees. Financial problems are the root cause of depression [45]. The high prevalence of depression in healthcare employees increased due to the advancing financial stressors (e.g., joblessness, low household income, poor savings). Given the statement, the research showed that COVID-19 financial stressors made 42.7% of individuals face symptoms of depression [48].

Considerably, financial wellness is crucial for achieving successful job performance. Employees’ financial health is the prime component determining the individual financial conditions [49]. Unfortunately, the global economic crises threatened the world’s financial structure during the pandemic, making individuals feel insecure about their employment [50]. The COVID-19 incremental layoffs lowered the individual work performance in the public and private service industry (e.g., hospitals). The unmanageable situation made employees face unfavorable financial conditions, thus impeding the employees’ job performance [51]. In particular, the COVID-19 economic consequences negatively influenced the psychological construct of the healthcare workers, hampering their work performance.

However, the height of the COVID-19 pandemic appeared to be strongly associated with anxiety. The financial distress during the pandemic made the healthcare workers worry about the susceptible financial condition. The pandemic imposing financial burdens impacts the mental health of the individuals, thus making them worry about their household finances. Accordingly, the study reveals a large population to report high anxiety (23.1%) due to the increasing financial concerns [52]. Perhaps, the COVID-19 susceptibility declared the worldwide depression, causing billions of people to lose their jobs. The high period of widespread effects increased job insecurity, thus affecting the individual mental health. Indeed, the great repression made the employees report colossal anxiety due to an increasing sense of job insecurity [53].

Weiss and Cropanzano [19] presented the affective event theory. Anything from getting reprimanded for bad work performance to receiving a free cup of coffee for a good performance might be either positive or negative. Work-related stressors, such as management styles and colleague behavior, can impact employees’ moods. Positive and unpleasant occurrences at work can cause long-term emotional reactions that affect how happy you are at work, how far you can go in your career, and how committed you are to the company [21].

Additionally, the changing economic conditions during the pandemic led the employees to experience financial stress, substantially worrying about their household expenses. Financial stress has a deep connection with employees’ family well-being. The COVID-19 economic downturn increased individuals’ financial hardship, thereby experiencing financial stress [54]. As such, it is found that:

**Hypothesis** **3** **(3a):** *Financial concerns have a positive and significant impact on depression*.

**Hypothesis** **3** **(3b):** *Financial concerns have a negative and significant impact on job performance*.

**Hypothesis** **3** **(3c):** *Financial concerns have a positive and significant impact on anxiety*.

**Hypothesis** **3** **(3d):** *Financial concerns have a positive and significant impact on stress*.

### 2.4. The Mediating Role of Depression

Depression, an unfavorable feeling of distress, brings unprecedented consequences, influencing the individual quality of life [55]. Indeed, depression was massively observed during the pandemic among the health workforce. The increased symptoms of COVID-19 made the global health sector the most vulnerable to experience severe symptoms of depression and distress [56]. Depression substantially impedes the work outcome (e.g., productivity, performance) [57]. The increased depression makes the employees feel exhausted and detached from the work [58], thus impacting their ability to provide premium quality service [59]. A health professional aims to provide high-quality service to the patients. Perhaps, for obtaining professional performance, the psychological well-being of the healthcare workers needs to be ensured [60].

Undoubtedly, depression is the root cause significantly influencing the workers’ functioning (i.e., job performance) [61]. Depression leads to poor mental functioning, making the employees incapable of performing work-related duties. In particular, psychological concerns manifest the symptoms of depression, thus hindering work performance [40]. Given the statement, the research on the healthcare professional shows that depression is a distinct phenomenon elevating feelings of dissatisfaction [62], thus making it difficult for individuals to cope with the increasing psychological problems. Ensuring work-related well-being is vital for enhancing job performance. In support, the findings indicated that during COVID-19, depressive symptoms elevated the psychological concern [63], leading to poor job performance [64].

In addition to psychological concerns, depressive symptoms were recorded in the healthcare workforce. Depression is associated with prolonged working hours and minimum salary. Socioeconomic factors determine the level of depression in the workers, thus influencing the work outcome [57]. The financial concerns exert financial distress on employees’ job performance. In response, the study shows that during COVID-19, the enhanced feeling of job insecurity among the doctors aggravated the symptoms of psychological depression, substantially affecting their job performance [65]. Therefore, it is found that:

**Hypothesis** **4:** *Depression negatively mediates the relation between COVID-19 fear, psychological concerns, financial concerns, and job performance*.

### 2.5. The Mediating Role of Anxiety

Over the years, researchers are picking up anxiety as the prime reason behind poor employee performance. Anxiety is a disrupting feeling that affects the individual mental functioning. Anxiety makes the employees worried about the job, potentially increasing the turnover rate. Anxiety influences the employees’ productivity, thus hindering their job performance. In support of the statement, the study indicates a significant negative relationship between anxiety and employees’ work performance [66].

In recent years, the coronavirus disease manifested intense fear among healthcare workers, leading to enduring anxiety. This intensified feeling of loneliness triggers the anxiety symptoms. The COVID-19 fear leads to a negative job attitude due to the high efficacy of the psychological distress (i.e., anxiety) [67]. Given the statement, the research shows that the COVID-19 pandemic illuminated anxiety symptoms among the employees, explicitly affecting their job performance [68]. COVID-19 anxiety impacts the individual quality of life, subsequently distressing the employees’ performance [69].

In particular, ensuring workplace safety has become the core concern of health professionals. The increased psychological issues regarding workers’ wellness have become a global public health problem. The COVID-19 psychological vulnerability generates negative catastrophic thoughts, thus leading to severe anxiety disorders [70]. The health-related concerns make individuals worry about their psychological well-being. Psychological deterioration makes the workers experience increased job anxiety, subsequently affecting the work outcomes [71]. Job-related worry diminishes job performance making it difficult for employees to fulfill the task requirement. During COVID-19, the emerging anxieties adversely influenced the employees’ expectations. Given the articulation, the research shows that the increasing job strain decreases the likelihood of meeting the job requirement (i.e., job performance) [42].

A significant risk associated with the COVID-19 psychological concerns backfires on the employees’ performance, leading to short-term monetary benefits. Consistently, coronavirus anxiety created severe worry for financial stability. Reported symptoms of the pandemic significantly influenced the employees’ mental health, thus calling the immediate response to increasing financial anxiety [72]. In response to the COVID-19 financial concerns, most of the world’s population experienced a threat to their finances due to the increasing economic consequences [73]. The financial strain associated with the mental disorder (e.g., anxiety) decreased the household income, making the employee face the emerging economic hardship [74]. This adjustment to the financial crisis is potentially associated with a high level of anxiety. The intense economic consequences (e.g., unemployment, layoffs, job insecurity) intensified the psychological distress [75], thus affecting the individual’s job performance [51]. Consequently, based on the above literature, we developed the following hypothesis:

**Hypothesis** **5:** *Anxiety negatively mediates the relation between COVID-19 fear, psychological concerns, financial concerns, and job performance*.

### 2.6. The Mediating Role of Stress

Over the years, several factors aggravated the stress symptoms among the frontline health representatives. The heavy workload, excessive work pressure, and enduring shifts significantly made the employee face numerous health challenges due to increased stress and anxiety. The work pressure makes the employees work less effectively, subsequently decreasing the individuals’ productivity and job quality. Job stress involves responding to environmental stimuli. Recent studies stated job stress to be the prime factor influencing individual work performance. Stress is negatively associated with an individual’s performance [76]. In particular, stress makes the individual negatively respond to work activities, thus achieving detrimental job performance [77]. Consistently, the study conducted in the American Dental Clinic Center shows that 108 employees experienced adverse effects of work stress affecting their job performance [78].

Considerably, the psychological reaction to COVID-19 suggests that the health vulnerabilities manifested extreme stress symptoms in healthcare employees. The health workers and other professionals have gone through traumatic stress, subsequently declining their work productivity. This decreased efficiency is due to the developing COVID-19 fear among the individuals [79]. In the account of COVID-19 fear, the study shows that work-related stress negatively affects employee performance, thus unfavorably influencing the working environment [78].

In particular, employees face stress if they do not meet the job obligation. The undesirable stress hinders the completion of their work task. Stress makes employees overburdened, subsequently minimizing their efforts in satisfying the job requirement. The intense distress and frustration magnify the feeling of powerlessness, making the employee incapable of performing the daily tasks. The psychological concerns made the health workers stressed about being infected, thus stopping the individual from performing well [80]. Indeed, the increasing health concerns during the pandemic prompted individuals’ desire for effective work performance, thereby fostering adverse psychological consequences (e.g., stress, anxiety, depression).

Poor mental health brings deleterious outcomes, thus making the individual vulnerable. [9]. Nurses are the frontline warriors that combat several psychological issues. The occupational workload poses a greater risk to the nurses’ profession, thereby detrimentally affecting their service efficiency. A recent study postulates an adverse effect of stress on employees’ (i.e., nurses’) work effectiveness and psychological well-being during the pandemic period. In explanation, the study shows that post-traumatic stress disorder made the nurses care about the lives of their loved ones, such as family, friends, and colleagues, thus driving the psychological distress to affect their performance [70].

In addition to threatening psychological health, COVID-19 tremendously disrupted the financial conditions of individuals while bringing economic crises [81]. Numerous factors accelerated the symptoms of stress among the healthcare staff. Besides the growing psychological issues, financial concerns made individuals stressed about their employment status (e.g., job security) [82]. The financial stress, loss of income, and inadequate job opportunities disrupted the employees’ lives while critically lowering their economic growth. Globally, the COVID-19 incident made many individuals bear the severe effect of financial loss along with the lack of basic needs [83]. Consequently, based on the prior literature, the hypothesis concludes the following.

**Hypothesis** **6:** *Stress negatively mediates the relation between COVID-19 fear, psychological concerns, financial concerns, and job performance*.

**Figure 1:**
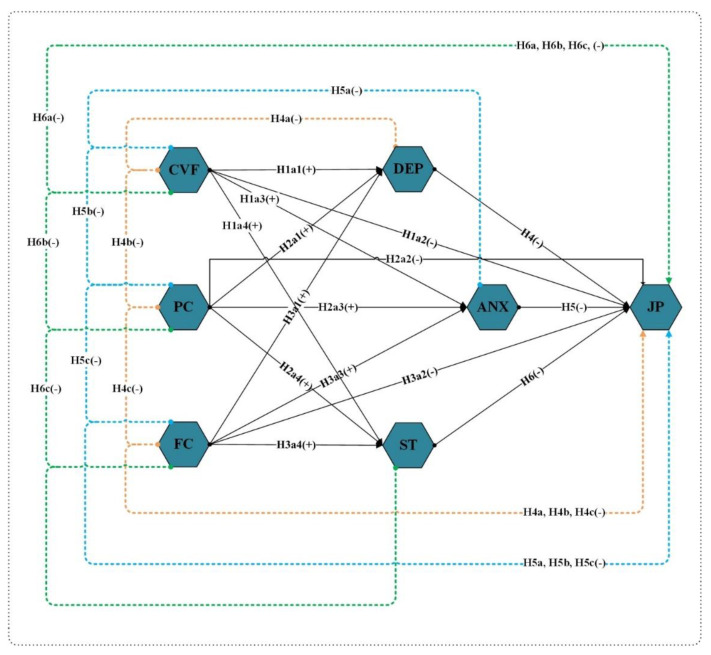
Conceptual Framework. CVF—Fear of COVID-19. PC—Psychological Concerns; FC—Financial Concerns; DEP—Depression; ANX—Anxiety; ST—Stress; and JP—Job Performance.

Figure 1 shows the study conceptual framework, which includes independent variables (Fear of COVID-19, psychological concerns, and financial concerns), mediating variables (depression; anxiety, stress), and dependent variable (employees’ job performance).

## 3. Methodology

### 3.1. Participants

The participants of this study were healthcare workers who work at hospitals in the major and most populated province of Pakistan (Punjab). Purposive sampling is known as the judgmental sampling approach in which the scholar selects participants of the entire population to contribute to the study based on their opinion [84]. COVID-19 patients are treated at the highest number of government facilities in Pakistan. Aside from that, the facility is equipped with every type of testing possible. Moreover, Punjab province was considered in this study because COVID-19 sufferers are most numerous in the Punjab province. Table 1 provides the complete details of the demographic characteristics of respondents who participated in this study. In this study, 489 healthcare workers participated, of which 233 were male and 256 were female.

### 3.2. Procedure

This study uses a quantitative approach to determine diverse and unique aspects of the new infectious disease (COVID-19). It has affected the mental and psychological health of healthcare workers and individuals. Purposive sampling is a non-probability sampling technique in which “mechanisms selected for the sample are picked based on the researcher’s judgment”. Scholars frequently feel that by employing sound judgment, they can acquire a representative sample and save time and money. The number of respondents is restricted, yet they can reflect the personality traits of the whole population [84,85,86]. The purposive sampling approach was used to collect data from healthcare workers. For instance, if the study’s entire population is one million, the sample size must be at least 384 people, according to Sekaran [80]. The cross-sectional survey method was applied in this study to collect essential subjective information from 489 healthcare workers from major public hospitals of Pakistan during the interval of 3 October 2021 to 30 December 2021. Google online questionnaires were distributed among healthcare workers through different channels such as WhatsApp and email. Employees’ prior consent was obtained before questionnaires’ distribution. The researchers also presented the printed QR code of the questionnaire, and the employee scanned the code to fill out the questionnaire. In this study, 570 online surveys were distributed, and 489 valid questionnaires were received. The response rate to valid filled questionnaires was 85.7%. All respondents voluntarily participated in this study and signed an informed consent form to exclude employees with a history of mental illness or failure to cooperate with expression. In this study, we applied Harman’s single-factor approach to check the common method bias (CMB). The variance extracted by one single factor is 17.228% which is less than 50%, indicating no common method bias in this study [87].

### 3.3. Measurement Scale

The study questionnaire was generally composed of three parts (e.g., study overview, variable-related questions, and demographic-characteristics-related questions). The current study adopted previously developed and tested variables items scale. Fear of COVID-19 was measured on the seven items scale adopted from Ahorsu et al. [88]. In the study of Ahorsu [88], the Cronbach’s alpha (α) value of Fear of COVID-19 was 0.822. The scale sample items included “I am most afraid of coronavirus-19”, “It makes me uncomfortable to think about coronavirus-19”, and “My hands become clammy when I think about coronavirus-19”. Psychological Concerns were measured on the seven items scale, and financial concerns were measured on the four items scale adopted from the study of Yu et al. [89]. In the study of Yu [89], the Cronbach’s alpha value of psychological concerns was 0.884, and the financial concerns Cronbach’s alpha value was 0.823. The mediating variables (Stress (α–0.90), Depression(α–0.920), and Anxiety (α–0.860)) were measured on the seven items scale adopted from the study of Vignola and Tucci [90]. The sample items include “I didn’t feel enthusiastic about anything”, “I didn’t experience any positive feelings”, “I found it difficult to relax”, and “I felt depressed and had no motivation”. The six items scale of employees’ job performance was adopted from the study of Liao and Chuang [91] and Snape and Redman [92]. The Cronbach’s alpha value of employee’s job performance was 0.910 in the original study. Employee job performance scale items include “My performance is still as good as it was before COVID-19”, “I have adequate competencies to carry out my work effectively”. All questionnaires were measured on a 5-point Likert scale ranging from 1 to 5, with 1 indicating strong disagreement and 5 indicating strong agreement. The regularly used multi-item questionnaire was developed to assess the study’s composition.

### 3.4. Statistical Analysis

The data were analyzed using the Smart PLS software (Version 3.3.7). The structural equation modeling (SEM) technique was applied, and confirmatory factor analysis (CFA) was conducted to determine the model’s internal validity and reliability [86]. Additionally, PLS-SEM was used to test the hypotheses developed between study variables (CVF, PC, FC, DEP, ST, ANX, and JP).

## 4. Results

Table 2 presents the mean and standard deviation values of each variable’s items. All the values are within range (Minimum 1 and Maximum 5).

### 4.1. Assessment of Measurement Model

We tested the measurement model’s reliability and validity in the first analysis phase. Model internal reliability was used to assess the internal consistency measure through “composite reliability” (CR) (see Table 3). The composite reliability varied from 0.871 to 0.918, higher than the usually accepted 0.70 criterion [93,94].

In addition, the construct validity of the assessments of the measurement model was assessed using convergent validity “average-variance-extracted” (AVE). The Fornell–Larcker criterion method was adopted to check the discriminant validity [95].

The AVE value varied between 0.573 to 0.651, indicating that convergent validity is acceptable, as indicated by Hair [96]. The score of AVE must not be considerably less than 0.5, and all factor items’ standardized factor loadings must not be significantly less than 0.5. Additionally, the square root of each variable AVE was defined to determine discriminant validity [97]. As illustrated in Table 3, the square root of each variable of the AVE value on the diagonal is more significant than the correlations between it and all other constructs in the study model.

Furthermore, the findings of the (Heterotrait–Monotrait Ratio) “HTMT” [98], a highly recommended approach for diagnosing discriminant validity, were less than the usually acknowledged threshold value of 0.85 [99], confirming the measurement of discriminant validity (see Table 4 and Table 5). Finally, these findings showed that common technique bias does not pose a risk in this study.

Table 6 shows the factor loadings values of all variables. The variable anxiety has a seven-items scale, and all the items have more than 0.7 values.

**Figure 2:**
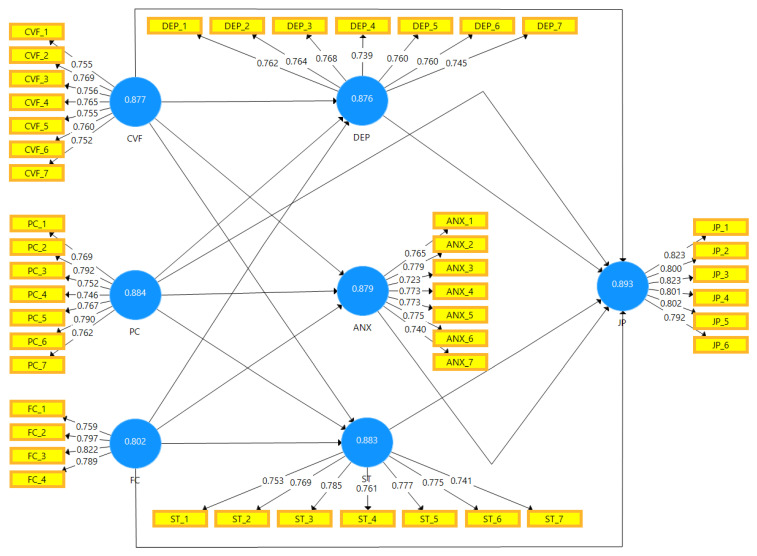
Graphical Representation of Assessment of Measurement Model.

Table 7 shows variance influence factor values of independent variables (Fear of COVID-19, psychological concerns, financial concerns), mediating variables (anxiety, depression, and stress), and the dependent variable (employees’ job performance). Figure 2 is the graphical representation of the assessment of the measurement model.

Table 8 shows the study model fit summary. As per Hu and Bentler [100], the standardized root mean square residual (SRMR) should be <0.08. In the current study, the SRMR saturated model value is 0.027, and the estimated model value is 0.036. Bentler and Bonnet [101] suggested that the Normed Fit Index (NFI) value should be >0.80. In our study, the NFI saturated model value was 0.914, and the NFI-estimated model is 0.910. All these values are within the range.

### 4.2. Structural Model

Table 9 results show that CVF (H1a1, H1a3, and H1a4) have a positive direct effect on DEP, ANX, and ST (β = 0.275, *p* < 0.001; β = 0.257, *p* < 0.0001; and β = 0.272, *p* < 0.0001, respectively). However, H1a2 showed that CVF has a negative direct effect on employees’ JP (β = −0.274, *p* < 0.0001). This means that high CVF results in low job performance of employees. Hypothesis (H2a1, H2a3, and H2a4) results showed that PC has a positive significant direct effect on DEP, ANX, and ST (β = 0.286, *p* < 0.0001; β = 0.296, *p* < 0.0001; and β = 0.266, *p* < 0.0001, respectively), but (H2a2) the finding showed that PC has a negative direct effect on employees’ JP (β = −0.106, *p* < 0.001). This means that high PC resulted in low job performance of employees. The hypothesis H3a1, H3a3, and H3a4 result revealed that that FC has a positive direct effect on DEP, ANX, and ST (β = 0.229, *p* < 0.0001; β = 0.238, *p* < 0.0001; and β = 0.255, *p* < 0.0001, respectively), whereas H3a2 showed a negative impact on employees’ JP (β = −0.254, *p* < 0.0001). This means that high FC resulted in low job performance of employees. Hypothesis H4 to H6 (DEP, ANX, and ST) showed a negative significant impact on employees’ JP (β = −0.191, *p* < 0.001; β = −0.105, *p* < 0.0001; and β = −0.145, *p* < 0.0001, respectively).

**Figure 3:**
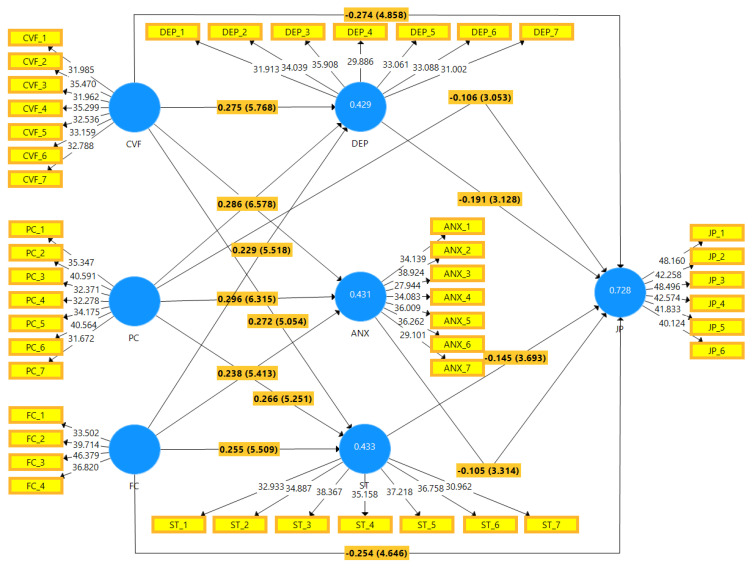
Graphical representation of the structural model.

As results show in Table 10, DEP mediates the relationship between CVF and JP (β = −0.053, *p* < 0.0001; H4a), and Hypothesis H4b findings revealed that DEP mediates the relationship between psychological concerns and job performance (β = −0.055, *p* < 0.0001). The H4c result showed that DEP mediates the relationship between FC and JP (β = −0.044, *p* < 0.0001). In addition, the results of hypothesis H5a showed that ANX mediates the relationship between CVF and JP (β = −0.027, *p* < 0.0001), and the results of hypothesis H5b showed that ANX mediates the relationship between PC and JP (β = −0.031, *p* < 0.0001). Hypothesis H5c, H5a states that ANX mediates the relationship between FC and JP (β = −0.025, *p* < 0.0001). Moreover, ST mediates the relationship between CVF and JP (β = −0.039, *p* < 0.0001; H6a), and ST mediates the relationship between PC and JP (β = −0.038, *p* < 0.0001; H6b). Hypothesis H6c showed the mediating role of ANX between FC and JP (β = −0.037, *p* < 0.0001). In support of the above mentioned, all hypotheses of both direct and indirect effect are significant and partially mediated (see Figure 3).

**Figure 4:**
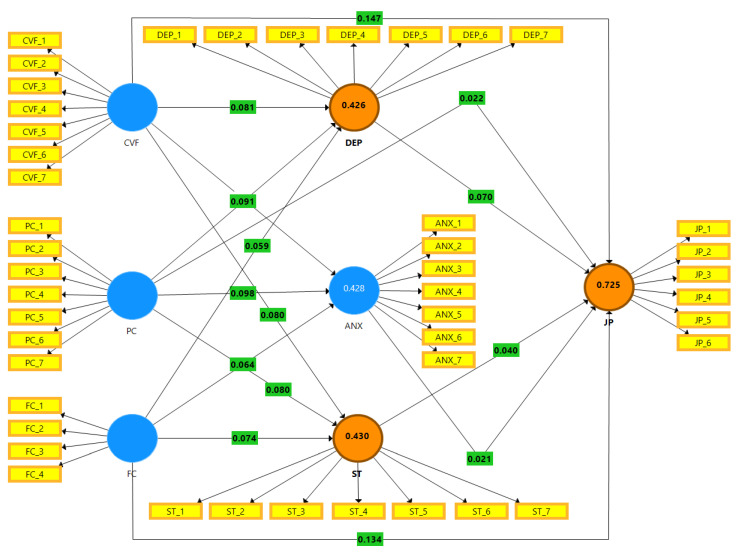
Graphical representation of R^2^ and f^2^.

Table 11 showed variables values of R^2^, f^2^, and Q^2^. The job performance adjusted R^2^ value is 0.725, anxiety (0.428), depression (0.426), and stress (0.430). The Q^2^ values of anxiety, depression, job performance, and stress are 0.227, 0.243, 0.469, and 0.251, respectively. Figure 4 is the graphical representation of R^2^ and f^2^ values.

## 5. Discussion

The COVID-19 unique effect has produced significant disruptions in the lives of frontline workers, thereby leading them to experience unprecedented psychological and financial repercussions. This pernicious calamity exposed individuals to a high level of psychiatric disorders, thus influencing their job performance. The previous literature shows that the pandemic forced the global economies to the edge of breaking, thus making the psychological aspect the vital concern of future researchers. The COVID-19 pandemic fostered economic repercussions, making most people experience difficulties meeting both ends. The prolonged continuality of the pandemic weakened the individual financial stability, thus declining their income and performance. In particular, this downturn of the COVID-19 raised several questions on the increased global economic volatility and psychological concerns in the world’s hospitability sector, thus negatively influencing the performance of the healthcare workers.

Empirically, this current study investigated the effect of COVID-19 Fear, psychological and financial concerns on individuals’ well-being and work performance. Indeed, Section 5 provides new insight into COVID-19 emergencies by discussing the study findings in the light of the previous research.

In recent times, the COVID-19 pandemic unexpectedly destroyed human society, thereby making individuals bear severe vulnerabilities of the pandemic. Given the articulation, the study shows that COVID-19 fear has increased the risk of developing psychological problems among healthcare employees [102], subsequently impeding their job performance [30]. Perhaps, based on the study findings, the hypotheses H1a1, H1a2, H1a3, and H1a4 are consistent with the previous literature that indicates that the increased COVID-19 susceptibility made employees report high symptoms of distress and anxiety [103], eventually influencing their work outcome.

Altogether, the COVID-19 hard-hit adversely affected the individual psychological well-being [104], thus fostering symptoms of fear and anxiety among individuals. In particular, this high coronavirus infectivity made the employees fearful of being infected [105]. Considerably, this research supports our findings, indicating that psychological concerns made individuals worry about their well-being. Indeed, this deadly widespread disease raised the psychological concern in healthcare workers, thus making them underperform their work tasks [106]. Hence, the prior research supports the current findings by significantly accepting the study hypotheses (i.e., H2b1, H2b2, H2b3, H2b4).

Furthermore, in addition to the emerging psychological concerns, this study also reports an increased financial worry among individuals. The results show that this widespread infection made the economic stress accelerate the symptoms of psychological disorders, thereby influencing the employees’ job performance. The global pandemic severely affected the financial well-being of most individuals, thus increasing the level of economic anxiety and fear in individuals’ lives [107]. Consistently, the study shows that the increasing financial volatility and instabilities strongly correlate with higher mental distress and psychiatric disorders (e.g., anxiety and stress) [108], ultimately impeding the employees’ work performance [51]. Hence, the study established a significant direct relationship between the variables. (i.e., H3c2, H3c3, H3c4), thus significantly corroborating the previous literature.

Indeed, the financial cost linked with psychological health is essential in understanding and improving healthcare. During the COVID-19 pandemic, the progressing psychological concerns immensely affected the employees’ work performance. In explaining this notion, the study showed that depression, mental distress, and anxiety heightened fear in frontline workers [109], potentially impeding their job performance [68]. In addition, COVID-19 vulnerabilities also raised financial worries in individuals. Accordingly, the study showed that during the COVID-19 pandemic, the progressing financial concerns (e.g., job insecurity) increased depression and anxiety in healthcare employees, negatively affecting their work performance [110]. Therefore, the prior studies showed that COVID-19 increasing vulnerabilities led employees to exhibit poor job performance. As a result, this study highlighted the role of the psychological factors (i.e., depression, anxiety, stress) in significantly mediating the relationship with COVID-19 fear, psychological concerns, financial concerns, and job performance. Considerably, based on the study findings, the results established a significant indirect relationship with accepting the study hypotheses (e.g., H4, H5, H6). Subsequently, this current study contributed to the significant finding on the healthcare workforce by providing an in-depth overview of the vulnerabilities caused by the pandemic crises.

In particular, this paper acknowledges the psychological disorders and recognizes the need for the healthcare workforce to mitigate the public health challenges during the pandemic period. The study provides valuable information to the health practitioners, policymakers, and future researchers about the public health emergencies exacerbating the negative effect on the individual’s mental health. It empowers the health workers to safeguard their mental health for ensuring effective performance. It explains the value of exploring self-care strategies through implementing protective measures against the increasing psychological and financial issues.

The current study also presents some limitations; the study’s first limitation is data collection from the healthcare works located only in the Punjab province of Pakistan. The study’s second limitation is the sample size. Future studies may consider some moderating variables related to the organization. The current study also can be replicated in cross-cultural countries. To conclude, the pandemic has posed considerable health challenges to worldwide industries while immensely threatening human civilization under the constraints of this devastating situation.

## 6. Conclusions

The study explains the effects of the pandemic, leading to increased psychological illness and economic concerns. For understanding the COVID-19 vulnerabilities, different emotions were targeted in the study, such as COVID-19 fear, depression, anxiety, and stress. Perhaps, this study provides a great significance by combining all the essential elements needed for gaining a clear picture of the devastating effects of the pandemic. In particular, the study findings show that this global picture of the unique calamity negatively influenced individuals’ psychological health. This catastrophic widespread disease aggravated the anxiety and fear in individuals, thus declining the performance of the health workforce. In addition to raising concerns about mental well-being, the results also show that individuals are worried about their career and financial status. These abrupt changes have ordinarily stimulated work productivity, thus realizing the need for mitigating the event challenges. Hence, this paper calls for effective pandemic management. Concerning the COVID-19 intensity, the study showcases the need for extensive healthcare services for ensuring the well-being of the individuals. Consequently, the impact of COVID-19 on psychological concerns, financial concerns, and job performance essentially requires designing effective nursing strategies for gaining better health outcomes.

## Figures and Tables

**Table 1:** Demographic Characteristics.

Items	Frequency (N = 489)	(%)	Mean
**Gender**			1520
Male	233	47.6	
Female	256	52.4	
**Age**			3090
19–30	54	11	
31–40	106	21.7	
41–50	134	27.4	
51–60	134	27.4	
>61	61	12.5	
**Marital Status**			1730
Single	134	27.4	
Married	355	72.6	
**Occupation**			2520
Doctors	66	13.5	
Nurses	187	38.2	
Technical	151	30.9	
Others	85	17.4	

**Table 2:** Descriptive Statistics.

Construct	Items	N	MIN	MAX	Mean	Std. Deviation
Fear of COVID-19	CVF_1	489	1	5	3.700	1.002
	CVF_2	489	1	5	3.620	0.966
	CVF_3	489	1	5	3.670	0.987
	CVF_4	489	1	5	3.710	0.995
	CVF_5	489	1	5	3.720	1.020
	CVF_6	489	1	5	3.760	1.026
	CVF_7	489	1	5	3.730	0.965
Psychological Concerns	PC_1	489	1	5	3.710	1.026
	PC_2	489	1	5	3.710	1.017
	PC_3	489	1	5	3.780	1.028
	PC_4	489	1	5	3.710	0.989
	PC_5	489	1	5	3.730	1.022
	PC_6	489	1	5	3.650	1.026
	PC_7	489	1	5	3.720	0.974
Financial Concerns	FC_1	489	1	5	3.730	0.988
	FC_2	489	1	5	3.690	0.999
	FC_3	489	1	5	3.720	1.056
	FC_4	489	1	5	3.720	0.976
Depression	DEP_1	489	1	5	3.700	1.024
	DEP_2	489	1	5	3.720	0.999
	DEP_3	489	1	5	3.750	1.003
	DEP_4	489	1	5	3.710	1.021
	DEP_5	489	1	5	3.620	1.004
	DEP_6	489	1	5	3.750	1.049
	DEP_7	489	1	5	3.730	1.007
Anxiety	ANX_1	489	1	5	3.680	1.001
	ANX_2	489	1	5	3.670	1.002
	ANX_3	489	1	5	3.730	0.980
	ANX_4	489	1	5	3.710	1.017
	ANX_5	489	1	5	3.720	0.985
	ANX_6	489	1	5	3.720	1.056
	ANX_7	489	1	5	3.700	1.005
Stress	ST_1	489	1	5	3.720	1.021
	ST_2	489	1	5	3.710	0.994
	ST_3	489	1	5	3.690	1.058
	ST_4	489	1	5	3.710	1.001
	ST_5	489	1	5	3.750	1.041
	ST_6	489	1	5	3.640	1.055
	ST_7	489	1	5	3.680	1.014
Job Performance	JP_1	489	1	5	2.300	1.081
	JP_2	489	1	5	2.320	1.061
	JP_3	489	1	5	2.310	1.057
	JP_4	489	1	5	2.340	1.037
	JP_5	489	1	5	2.310	1.041
	JP_6	489	1	5	2.370	1.055

**Table 3:** Reliability and Validity Analysis.

Construct	Items	Loading	α	CR	AVE
Fear of COVID-19	CVF_1	0.755	0.877	0.905	0.576
	CVF_2	0.769			
	CVF_3	0.756			
	CVF_4	0.765			
	CVF_5	0.755			
	CVF_6	0.760			
	CVF_7	0.752			
Psychological concerns	PC_1	0.769	0.884	0.910	0.591
	PC_2	0.792			
	PC_3	0.752			
	PC_4	0.746			
	PC_5	0.767			
	PC_6	0.790			
	PC_7	0.762			
Financial concerns	FC_1	0.759	0.802	0.871	0.628
	FC_2	0.797			
	FC_3	0.822			
	FC_4	0.789			
Depression	DEP_1	0.762	0.876	0.904	0.573
	DEP_2	0.764			
	DEP_3	0.768			
	DEP_4	0.739			
	DEP_5	0.760			
	DEP_6	0.760			
	DEP_7	0.745			
Anxiety	ANX_1	0.765	0.879	0.906	0.580
	ANX_2	0.779			
	ANX_3	0.723			
	ANX_4	0.773			
	ANX_5	0.773			
	ANX_6	0.775			
	ANX_7	0.740			
Stress	ST_1	0.753	0.883	0.909	0.587
	ST_2	0.769			
	ST_3	0.785			
	ST_4	0.761			
	ST_5	0.777			
	ST_6	0.775			
	ST_7	0.741			
Job performance	JP_1	0.823	0.893	0.918	0.651
	JP_2	0.800			
	JP_3	0.823			
	JP_4	0.801			
	JP_5	0.802			
	JP_6	0.792			

**Table 4:** Discriminant Validity Analysis (Fornel–Larcker).

Constructs	1	2	3	4	5	6	7
ANX	0.761						
CVF	0.546	0.759					
DEP	0.575	0.553	0.757				
FC	0.528	0.538	0.523	0.792			
JP	−0.639	−0.712	−0.675	−0.689	0.807		
PC	0.558	0.542	0.552	0.513	−0.628	0.768	
ST	0.559	0.554	0.555	0.538	−0.656	0.545	0.766

Note: Values on the diagonal represent the square root of the average variance extracted, while the off diagonals are correlations.

**Table 5:** Discriminant Validity Analysis (HTMT).

Constructs	1	2	3	4	5	6	7
ANX							
CVF	0.618						
DEP	0.653	0.63					
FC	0.627	0.641	0.623				
JP	0.718	0.804	0.763	0.815			
PC	0.63	0.615	0.626	0.609	0.706		
ST	0.633	0.626	0.629	0.638	0.736	0.615	

**Table 6:** Discriminant Validity Analysis (Cross-Loadings).

Construct’s Items	ANX	CVF	DEP	FC	JP	PC	ST
ANX_1	0.765	0.395	0.465	0.398	−0.475	0.444	0.431
ANX_2	0.779	0.443	0.470	0.433	−0.557	0.447	0.403
ANX_3	0.723	0.375	0.418	0.338	−0.433	0.375	0.403
ANX_4	0.773	0.405	0.424	0.372	−0.450	0.442	0.415
ANX_5	0.773	0.434	0.442	0.455	−0.503	0.439	0.460
ANX_6	0.775	0.469	0.464	0.421	−0.500	0.430	0.472
ANX_7	0.740	0.378	0.375	0.386	−0.474	0.388	0.394
CVF_1	0.393	0.755	0.423	0.408	−0.540	0.403	0.402
CVF_2	0.428	0.769	0.428	0.389	−0.550	0.435	0.421
CVF_3	0.379	0.756	0.389	0.417	−0.516	0.425	0.428
CVF_4	0.402	0.765	0.420	0.400	−0.544	0.402	0.407
CVF_5	0.423	0.755	0.437	0.398	−0.560	0.427	0.427
CVF_6	0.489	0.760	0.414	0.424	−0.537	0.399	0.443
CVF_7	0.379	0.752	0.423	0.423	−0.533	0.389	0.411
DEP_1	0.409	0.422	0.762	0.374	−0.481	0.431	0.423
DEP_2	0.446	0.382	0.764	0.369	−0.532	0.405	0.397
DEP_3	0.507	0.446	0.768	0.471	−0.529	0.451	0.459
DEP_4	0.418	0.410	0.739	0.376	−0.469	0.402	0.405
DEP_5	0.406	0.440	0.760	0.372	−0.505	0.416	0.410
DEP_6	0.451	0.415	0.760	0.416	−0.524	0.436	0.450
DEP_7	0.403	0.411	0.745	0.386	−0.532	0.381	0.394
FC_1	0.389	0.387	0.415	0.759	−0.542	0.408	0.423
FC_2	0.401	0.453	0.416	0.797	−0.570	0.386	0.427
FC_3	0.429	0.449	0.436	0.822	−0.547	0.426	0.455
FC_4	0.455	0.414	0.390	0.789	−0.527	0.406	0.401
JP_1	−0.486	−0.570	−0.533	−0.552	0.823	−0.502	−0.501
JP_2	−0.498	−0.566	−0.560	−0.532	0.800	−0.519	−0.500
JP_3	−0.546	−0.577	−0.576	−0.580	0.823	−0.512	−0.580
JP_4	−0.506	−0.583	−0.525	−0.571	0.801	−0.508	−0.538
JP_5	−0.524	−0.556	−0.557	−0.540	0.802	−0.494	−0.560
JP_6	−0.533	−0.593	−0.516	−0.561	0.792	−0.506	−0.493
PC_1	0.407	0.412	0.440	0.354	−0.468	0.769	0.407
PC_2	0.452	0.435	0.442	0.453	−0.504	0.792	0.419
PC_3	0.425	0.435	0.439	0.384	−0.481	0.752	0.413
PC_4	0.439	0.423	0.422	0.412	−0.516	0.746	0.429
PC_5	0.411	0.398	0.385	0.373	−0.456	0.767	0.445
PC_6	0.472	0.437	0.438	0.384	−0.510	0.790	0.424
PC_7	0.386	0.372	0.400	0.399	−0.437	0.762	0.391
ST_1	0.436	0.423	0.427	0.404	−0.510	0.411	0.753
ST_2	0.397	0.389	0.391	0.399	−0.481	0.368	0.769
ST_3	0.466	0.466	0.463	0.462	−0.530	0.482	0.785
ST_4	0.408	0.405	0.391	0.380	−0.475	0.424	0.761
ST_5	0.446	0.435	0.433	0.386	−0.498	0.411	0.777
ST_6	0.440	0.465	0.439	0.448	−0.553	0.419	0.775
ST_7	0.398	0.375	0.427	0.400	−0.462	0.400	0.741

**Table 7:** Variance Influence Factor.

Constructs	ANX	CVF	DEP	FC	JP	PC	ST
ANX					1.942		
CVF	1.628		1.628		1.887		1.628
DEP					1.930		
FC	1.56		1.56		1.770		1.560
JP							
PC	1.571		1.571		1.859		1.571
ST					1.913		

**Table 8:** Model Fit.

	Fit Criteria	Saturated Model	Estimated Model
SRMR	<0.08 (Hu and Bentler) [100]	0.027	0.036
NFI	>0.80 (Bentler and Bonnet) [101]	0.914	0.910
Chi-Square		1012.751	0.91

**Table 9:** Hypotheses testing Direct Effect.

Hypothesis	Direct	Std.	Std.	T	*p*
	Relationships	Beta	Error	Values	Values
H1a1	CVF→DEP	0.275	0.049	5.666	**
H1a2	CVF→JP	−0.274	0.056	4.869	***
H1a3	CVF→ANX	0.257	0.05	5.149	***
H1a4	CVF→ST	0.272	0.054	5.064	***
H2a1	PC→DEP	0.286	0.044	6.444	***
H2a2	PC→JP	−0.106	0.034	3.134	**
H2a3	PC→ANX	0.296	0.048	6.155	***
H2a4	PC→ST	0.266	0.051	5.189	***
H3a1	FC→DEP	0.229	0.042	5.508	***
H3a2	FC→JP	−0.254	0.056	4.532	***
H3a3	FC→ANX	0.238	0.044	5.439	***
H3a4	FC→ST	0.255	0.046	5.613	***
H4	DEP→JP	−0.191	0.061	3.146	**
H5	ANX→JP	−0.105	0.032	3.275	***
H6	ST→JP	−0.145	0.039	3.718	***

Note: ** *p* < 0.01, *** *p* < 0.001.

**Table 10:** Hypotheses testing Mediation Effect.

Hypothesis	Indirect	Std.	Std.	T	*p*
	Relationships	Beta	Error	Values	Values
H4a	CVF→DEP→JP	−0.053	0.019	2.757	**
H4b	PC→DEP→JP	−0.055	0.019	2.896	**
H4c	FC→DEP→JP	−0.044	0.017	2.537	*
H5a	CVF→ANX→JP	−0.027	0.01	2.586	*
H5b	PC→ANX→JP	−0.031	0.011	2.809	**
H5c	FC→ANX→JP	−0.025	0.01	2.607	**
H6a	CVF→ST→JP	−0.039	0.014	2.815	**
H6b	PC→ST→JP	−0.038	0.013	2.877	**
H6c	FC→ST→JP	−0.037	0.013	2.829	**

Note: * *p* < 0.05, ** *p* < 0.01, NS = not significant.

**Table 11:** Quality Criteria (R^2^, f^2^, and Q^2^).

Latent Variables	R^2^	R^2Adj^	Q^2^	f^2^
ANX	0.431	0.428	0.247	
DEP	0.429	0.426	0.243	
JP	0.728	0.725	0.469	
ST	0.433	0.430	0.251	
ANX→JP				0.021
CVF→ANX				0.072
CVF→DEP				0.081
CVF→JP				0.147
CVF→ST				0.080
DEP→JP				0.070
FC→ANX				0.064
FC→DEP				0.059
FC→JP				0.134
FC→ST				0.074
PC→ANX				0.098
PC→DEP				0.091
PC→JP				0.022
PC→ST				0.080
ST→JP				0.040

## Data Availability

The current study data can be obtained from the corresponding author.

## References

[1] Silva P.C.L., Batista P.V.C., Lima H.S., Alves M.A., Guimarães F.G., Silva R.C.P. (2020). COVID-ABS: An agent-based model of COVID-19 epidemic to simulate health and economic effects of social distancing interventions. Chaos Solitons Fractals.

[2] Rothan H.A., Byrareddy S.N. (2020). The epidemiology and pathogenesis of coronavirus disease (COVID-19) outbreak. J. Autoimmun..

[3] Pakpour A., Griffiths M. (2020). The fear of COVID-19 and its role in preventive behaviors. J. Concurr. Disord..

[4] Nicola M., Alsafi Z., Sohrabi C., Kerwan A., Al-Jabir A., Iosifidis C., Agha M., Agha R. (2020). The socio-economic implications of the coronavirus pandemic (COVID-19): A review. Int. J. Surg..

[5] Badahdah A., Khamis F., Al Mahyijari N., Al Balushi M., Al Hatmi H., Al Salmi I., Albulushi Z., Al Noomani J. (2021). The mental health of health care workers in Oman during the COVID-19 pandemic. Int. J. Soc. Psychiatry.

[6] (2004). WHO Promoting Mental Health: Concepts, Emerging Evidence.

[7] Shareef F., Chaudhary I.S., Azid T., Zafar M.R. (2020). Does Trade Openness Transfer Pollution across Borders: An Experience of Pakistan. Rev. Econ. Dev. Stud..

[8] Muller A.E., Hafstad E.V., Himmels J.P.W., Smedslund G., Flottorp S., Stensland S.Ø., Stroobants S., Van de Velde S., Vist G.E. (2020). The mental health impact of the covid-19 pandemic on healthcare workers, and interventions to help them: A rapid systematic review. Psychiatry Res..

[9] Lei L., Huang X., Zhang S., Yang J., Yang L., Xu M. (2020). Comparison of Prevalence and Associated Factors of Anxiety and Depression Among People Affected by versus People Unaffected by Quarantine During the COVID-19 Epidemic in Southwestern China. Med. Sci. Monit..

[10] Mamun M.A., Sakib N., Gozal D., Bhuiyan A.I., Hossain S., Bodrud-Doza M., Al Mamun F., Hosen I., Safiq M.B., Abdullah A.H. (2021). The COVID-19 pandemic and serious psychological consequences in Bangladesh: A population-based nationwide study. J. Affect. Disord..

[11] Huang Y., Zhao N. (2020). Generalized anxiety disorder, depressive symptoms and sleep quality during COVID-19 outbreak in China: A web-based cross-sectional survey. Psychiatry Res..

[12] Kang L., Li Y., Hu S., Chen M., Yang C., Yang B.X., Wang Y., Hu J., Lai J., Ma X. (2020). The mental health of medical workers in Wuhan, China dealing with the 2019 novel coronavirus. Lancet Psychiatry.

[13] Imran N., Masood H.M.U., Ayub M., Gondal K.M. (2020). Psychological impact of COVID-19 pandemic on postgraduate trainees: A cross-sectional survey. Postgrad. Med. J..

[14] Thayer Z.M., Gildner T.E. (2021). COVID-19-related financial stress associated with higher likelihood of depression among pregnant women living in the United States. Am. J. Hum. Biol..

[15] Tu Y., Li D., Wang H.-J. (2021). COVID-19-induced layoff, survivors’ COVID-19-related stress and performance in hospitality industry: The moderating role of social support. Int. J. Hosp. Manag..

[16] Abbas M., Malik M., Sarwat N. (2021). Consequences of job insecurity for hospitality workers amid COVID-19 pandemic: Does social support help?. J. Hosp. Mark. Manag..

[17] Baert S., Lippens L., Moens E., Sterkens P., Weytjens J. (2020). How Do We Think the COVID-19 Crisis Will Affect Our Careers.

[18] Søvold L.E., Naslund J.A., Kousoulis A.A., Saxena S., Qoronfleh M.W., Grobler C., Münter L. (2021). Prioritizing the Mental Health and Well-Being of Healthcare Workers: An Urgent Global Public Health Priority. Front. Public Health.

[19] Weiss H.M., Suckow K., Cropanzano R. (1999). Effects of justice conditions on discrete emotions. J. Appl. Psychol..

[20] Martocchio J.J., Jimeno D.I. (2003). Employee absenteeism as an affective event. Hum. Resour. Manag. Rev..

[21] Weiss H.M., Beal D.J. (2005). Reflections on affective events theory. The Effect of Affect in Organizational Settings.

[22] Cropanzano R., Dasborough M.T., Weiss H.M. (2017). Affective events and the development of leader-member exchange. Acad. Manag. Rev..

[23] Bay E.J., Algase D.L. (1999). Fear and Anxiety: A Simultaneous Concept Analysis. Int. J. Nurs. Terminol. Classif..

[24] Dong L., Freedman V.A., Mendes de Leon C.F. (2020). The Association of Comorbid Depression and Anxiety Symptoms With Disability Onset in Older Adults. Psychosom. Med..

[25] Asmundson G.J.G., Taylor S. (2020). Coronaphobia: Fear and the 2019-nCoV outbreak. J. Anxiety Disord..

[26] Tang F., Liang J., Zhang H., Kelifa M.M., He Q., Wang P. (2021). COVID-19 related depression and anxiety among quarantined respondents. Psychol. Health.

[27] Luo M., Guo L., Yu M., Jiang W., Wang H. (2020). The psychological and mental impact of coronavirus disease 2019 (COVID-19) on medical staff and general public – A systematic review and meta-analysis. Psychiatry Res..

[28] Sasaki N., Kuroda R., Tsuno K., Kawakami N. (2020). Workplace responses to COVID-19 associated with mental health and work performance of employees in Japan. J. Occup. Health.

[29] ERER B. (2020). Impact of Covid-19 Fear on Employee Performance. Soc. Sci..

[30] Ota I., Asada Y. (2020). The impact of preoperative screening system on head and neck cancer surgery during the COVID-19 pandemic: Recommendations from the nationwide survey in Japan. Auris Nasus Larynx.

[31] Soraci P., Ferrari A., Abbiati F.A., Del Fante E., De Pace R., Urso A., Griffiths M.D. (2020). Validation and Psychometric Evaluation of the Italian Version of the Fear of COVID-19 Scale. Int. J. Ment. Health Addict..

[32] Mertens G., Gerritsen L., Duijndam S., Salemink E., Engelhard I.M. (2020). Fear of the coronavirus (COVID-19): Predictors in an online study conducted in March 2020. J. Anxiety Disord..

[33] Silverman M.N., Heim C.M., Nater U.M., Marques A.H., Sternberg E.M. (2010). Neuroendocrine and Immune Contributors to Fatigue. PMR.

[34] Cohrdes C., Yenikent S., Wu J., Ghanem B., Franco-Salvador M., Vogelgesang F. (2021). Indications of Depressive Symptoms During the COVID-19 Pandemic in Germany: Comparison of National Survey and Twitter Data. JMIR Ment. Health.

[35] Mazza C., Ricci E., Biondi S., Colasanti M., Ferracuti S., Napoli C., Roma P. (2020). A Nationwide Survey of Psychological Distress among Italian People during the COVID-19 Pandemic: Immediate Psychological Responses and Associated Factors. Int. J. Environ. Res. Public Health.

[36] Wang C., Pan R., Wan X., Tan Y., Xu L., Ho C.S., Ho R.C. (2020). Immediate Psychological Responses and Associated Factors during the Initial Stage of the 2019 Coronavirus Disease (COVID-19) Epidemic among the General Population in China. Int. J. Environ. Res. Public Health.

[37] Schonfeld I.S., Verkuilen J., Bianchi R. (2019). Inquiry into the correlation between burnout and depression. J. Occup. Health Psychol..

[38] Nasharudin N.A., Idris M.A., Loh M.Y., Tuckey M. (2020). The role of psychological detachment in burnout and depression: A longitudinal study of Malaysian workers. Scand. J. Psychol..

[39] Presti G., McHugh L., Gloster A., Karekla M., Hayes S.C. (2020). The dynamics of fear at the time of COVID-19: A contextual behavioral science perspective. Clin. Neuropsychiatry.

[40] Hennekam S., Richard S., Grima F. (2020). Coping with mental health conditions at work and its impact on self-perceived job performance. Empl. Relat. Int. J..

[41] Daly M., Robinson E. (2021). Psychological distress and adaptation to the COVID-19 crisis in the United States. J. Psychiatr. Res..

[42] Clercq D., Azeem M.U., Haq I.U. (2020). But they promised! How psychological contracts influence the impact of felt violations on job-related anxiety and performance. Pers. Rev..

[43] Li Y., Scherer N., Felix L., Kuper H. (2021). Prevalence of depression, anxiety and post-traumatic stress disorder in health care workers during the COVID-19 pandemic: A systematic review and meta-analysis. PLoS ONE.

[44] Ismail H.N., Karkoulian S., Kertechian S.K. (2019). Which personal values matter most? Job performance and job satisfaction across job categories. Int. J. Organ. Anal..

[45] Hees S., Siewe Fodjo J.N., Wijtvliet V., Van den Bergh R., Faria de Moura Villela E., da Silva C.F., Weckhuysen S., Colebunders R. (2020). Access to healthcare and prevalence of anxiety and depression in persons with epilepsy during the COVID-19 pandemic: A multicountry online survey. Epilepsy Behav..

[46] Baud D., Qi X., Nielsen-Saines K., Musso D., Pomar L., Favre G. (2020). Real estimates of mortality following COVID-19 infection. Lancet Infect. Dis..

[47] Pappa S., Ntella V., Giannakas T., Giannakoulis V.G., Papoutsi E., Katsaounou P. (2020). Prevalence of depression, anxiety, and insomnia among healthcare workers during the COVID-19 pandemic: A systematic review and meta-analysis. Brain. Behav. Immun..

[48] Ettman C.K., Abdalla S.M., Cohen G.H., Sampson L., Vivier P.M., Galea S. (2020). Prevalence of Depression Symptoms in US Adults Before and During the COVID-19 Pandemic. JAMA Netw. Open.

[49] Narges D., Laily H.P. (2011). Determinants of financial wellness among Malaysia workers. African J. Bus. Manag..

[50] Yin J., Ni Y. (2021). COVID-19 event strength, psychological safety, and avoidance coping behaviors for employees in the tourism industry. J. Hosp. Tour. Manag..

[51] Chirumbolo A., Callea A., Urbini F. (2020). Job insecurity and performance in public and private sectors: A moderated mediation model. J. Organ. Eff. People Perform..

[52] Solomou I., Constantinidou F. (2020). Prevalence and Predictors of Anxiety and Depression Symptoms during the COVID-19 Pandemic and Compliance with Precautionary Measures: Age and Sex Matter. Int. J. Environ. Res. Public Health.

[53] Forbes M.K., Krueger R.F. (2019). The Great Recession and Mental Health in the United States. Clin. Psychol. Sci..

[54] Gassman-Pines A., Schenck-Fontaine A. (2019). Economic strain and job loss. APA Handbook of Contemporary Family Psychology: Applications and Broad Impact of Family Psychology (Volume 2).

[55] Bakhshi E., Morad A., Naderi M.R., Kalantari R. (2018). Associations of the Quality of Work Life and Depression, Anxiety, and Stress in the Employees of Healthcare Systems. J. Patient Saf. Qual. Improv..

[56] Gao J., Zheng P., Jia Y., Chen H., Mao Y., Chen S., Wang Y., Fu H., Dai J. (2020). Mental health problems and social media exposure during COVID-19 outbreak. PLoS ONE.

[57] Parent-Lamarche A., Marchand A., Saade S. (2020). Does Depression Mediate the Effect of Work Organization Conditions on Job Performance?. J. Occup. Environ. Med..

[58] Bianchi R., Verkuilen J., Schonfeld I.S., Hakanen J.J., Jansson-Fröjmark M., Manzano-García G., Laurent E., Meier L.L. (2021). Is Burnout a Depressive Condition? A 14-Sample Meta-Analytic and Bifactor Analytic Study. Clin. Psychol. Sci..

[59] Abdel Hadi S., Bakker A.B., Häusser J.A. (2021). The role of leisure crafting for emotional exhaustion in telework during the COVID-19 pandemic. Anxiety Stress Coping.

[60] Lohmann J., Shulenbayev O., Wilhelm D., Muula A.S., De Allegri M. (2019). Psychological wellbeing in a resource-limited work environment: Examining levels and determinants among health workers in rural Malawi. Hum. Resour. Health.

[61] Heath C., Sommerfield A., von Ungern-Sternberg B.S. (2020). Resilience strategies to manage psychological distress among healthcare workers during the COVID-19 pandemic: A narrative review. Anaesthesia.

[62] Extremera N., Mérida-López S., Quintana-Orts C., Rey L. (2020). On the association between job dissatisfaction and employee’s mental health problems: Does emotional regulation ability buffer the link?. Pers. Individ. Dif..

[63] Nelson B.W., Pettitt A.K., Flannery J.E., Allen N.B. (2020). Rapid Assessment of Psychological and Epidemiological Correlates of COVID-19 Concern, Financial Strain, and Health-Related Behavior Change in a Large Online Sample. PLoS ONE.

[64] Rodríguez-Socarrás M., Vasquez J.L., Uvin P., Skjold-Kingo P., Rivas J.G. (2018). Fatigue syndrome: Stress, Burnout and depression in Urology. Arch. Esp. Urol..

[65] Gasparro R., Scandurra C., Maldonato N.M., Dolce P., Bochicchio V., Valletta A., Sammartino G., Sammartino P., Mariniello M., di Lauro A.E. (2020). Perceived Job Insecurity and Depressive Symptoms among Italian Dentists: The Moderating Role of Fear of COVID-19. Int. J. Environ. Res. Public Health.

[66] Yokozawa K., Nguyen H.A., Tran T.B.H. (2021). Role of personal anxiety in individual kaizen behaviour and performance: Evidence from Japan. Int. J. Oper. Prod. Manag..

[67] Rajabimajd N., Alimoradi Z., Griffiths M. (2021). Impact of COVID-19-related fear and anxiety on job attributes: A systematic review. Asian J. Soc. Health Behav..

[68] Labrague L.J., Santos J.A.A. (2020). COVID-19 anxiety among front-line nurses: Predictive role of organisational support, personal resilience and social support. J. Nurs. Manag..

[69] Kumar P., Kumar N., Aggarwal P., Yeap J.A.L. (2021). Working in lockdown: The relationship between COVID-19 induced work stressors, job performance, distress, and life satisfaction. Curr. Psychol..

[70] Lai J., Ma S., Wang Y., Cai Z., Hu J., Wei N., Wu J., Du H., Chen T., Li R. (2020). Factors Associated With Mental Health Outcomes Among Health Care Workers Exposed to Coronavirus Disease 2019. JAMA Netw. Open.

[71] Fu S.Q., Greco L.M., Lennard A.C., Dimotakis N. (2021). Anxiety responses to the unfolding COVID-19 crisis: Patterns of change in the experience of prolonged exposure to stressors. J. Appl. Psychol..

[72] Torales J., O’Higgins M., Castaldelli-Maia J.M., Ventriglio A. (2020). The outbreak of COVID-19 coronavirus and its impact on global mental health. Int. J. Soc. Psychiatry.

[73] Baldwin R., Mauro B.W. (2020). Di Economics in the Time of COVID-19.

[74] Mann F.D., Krueger R.F., Vohs K.D. (2020). Personal economic anxiety in response to COVID-19. Pers. Individ. Dif..

[75] Wilson J.M., Lee J., Fitzgerald H.N., Oosterhoff B., Sevi B., Shook N.J. (2020). Job Insecurity and Financial Concern During the COVID-19 Pandemic Are Associated With Worse Mental Health. J. Occup. Environ. Med..

[76] Foy T., Dwyer R.J., Nafarrete R., Hammoud M.S.S., Rockett P. (2019). Managing job performance, social support and work-life conflict to reduce workplace stress. Int. J. Product. Perform. Manag..

[77] Deng J., Guo Y., Ma T., Yang T., Tian X. (2019). How job stress influences job performance among Chinese healthcare workers: A cross-sectional study. Environ. Health Prev. Med..

[78] Yunita P.I., Saputra I.G.N.W.H. (2019). Millennial generation in accepting mutations: Impact on work stress and employee performance. Int. J. Soc. Sci. Humanit..

[79] Sekowski M., Gambin M., Hansen K., Holas P., Hyniewska S., Wyszomirska J., Pluta A., Sobańska M., Łojek E. (2021). Risk of Developing Post-traumatic Stress Disorder in Severe COVID-19 Survivors, Their Families and Frontline Healthcare Workers: What Should Mental Health Specialists Prepare For?. Front. Psychiatry.

[80] Kapata N., Ihekweazu C., Ntoumi F., Raji T., Chanda-Kapata P., Mwaba P., Mukonka V., Bates M., Tembo J., Corman V. (2020). Is Africa prepared for tackling the COVID-19 (SARS-CoV-2) epidemic. Lessons from past outbreaks, ongoing pan-African public health efforts, and implications for the future. Int. J. Infect. Dis..

[81] Haider I.I., Tiwana F., Tahir S.M. (2020). Impact of the COVID-19 Pandemic on Adult Mental Health. Pakistan J. Med. Sci..

[82] Rossell S.L., Neill E., Phillipou A., Tan E.J., Toh W.L., Van Rheenen T.E., Meyer D. (2021). An overview of current mental health in the general population of Australia during the COVID-19 pandemic: Results from the COLLATE project. Psychiatry Res..

[83] Gloster A.T., Lamnisos D., Lubenko J., Presti G., Squatrito V., Constantinou M., Nicolaou C., Papacostas S., Aydın G., Chong Y.Y. (2020). Impact of COVID-19 pandemic on mental health: An international study. PLoS ONE.

[84] Campbell S., Greenwood M., Prior S., Shearer T., Walkem K., Young S., Bywaters D., Walker K. (2020). Purposive sampling: Complex or simple? Research case examples. J. Res. Nurs..

[85] Sharma G. (2017). Pros and cons of different sampling techniques. Int. J. Appl. Res..

[86] Etikan I., Bala K. (2017). Sampling and sampling methods. Biom. Biostat. Int. J..

[87] Podsakoff P.M., MacKenzie S.B., Lee J.-Y., Podsakoff N.P. (2003). Common method biases in behavioral research: A critical review of the literature and recommended remedies. J. Appl. Psychol..

[88] Ahorsu D.K., Lin C.-Y., Imani V., Saffari M., Griffiths M.D., Pakpour A.H. (2020). The fear of COVID-19 scale: Development and initial validation. Int. J. Ment. Health Addict..

[89] Yu J., Park J., Hyun S.S. (2021). Impacts of the COVID-19 pandemic on employees’ work stress, well-being, mental health, organizational citizenship behavior, and employee-customer identification. J. Hosp. Mark. Manag..

[90] Vignola R.C.B., Tucci A.M. (2014). Adaptation and validation of the depression, anxiety and stress scale (DASS) to Brazilian Portuguese. J. Affect. Disord..

[91] Liao H., Chuang A. (2004). A multilevel investigation of factors influencing employee service performance and customer outcomes. Acad. Manag. J..

[92] Snape E., Redman T. (2010). HRM practices, organizational citizenship behaviour, and performance: A multi-level analysis. J. Manag. Stud..

[93] Asghar M., Gull N., Tayyab M., Zhijie S., Tao X. (2020). Polychronicity at work: Work engagement as a mediator of the relationships between job outcomes. J. Hosp. Tour. Manag..

[94] Carrión G.C., Nitzl C., Roldán J.L. (2017). Mediation analyses in partial least squares structural equation modeling: Guidelines and empirical examples. Partial Least Squares Path Modeling.

[95] Mikalef P., Pateli A. (2017). Information technology-enabled dynamic capabilities and their indirect effect on competitive performance: Findings from PLS-SEM and fsQCA. J. Bus. Res..

[96] Hair J.F., Risher J.J., Sarstedt M., Ringle C.M. (2019). When to use and how to report the results of PLS-SEM. Eur. Bus. Rev..

[97] Fornell C., Larcker D.F. (1981). Evaluating structural equation models with unobservable variables and measurement error. J. Mark. Res..

[98] Ab Hamid M.R., Sami W., Sidek M.H.M. (2017). Discriminant validity assessment: Use of Fornell & Larcker criterion versus HTMT criterion. J. Phys. Conf. Ser..

[99] Hair J.F., Hult G.T.M., Ringle C.M., Sarstedt M., Danks N.P., Ray S. (2021). An introduction to structural equation modeling. Partial Least Squares Structural Equation Modeling (PLS-SEM) Using R.

[100] Hu L., Bentler P.M. (1999). Cutoff criteria for fit indexes in covariance structure analysis: Conventional criteria versus new alternatives. Struct. Equ. Model. Multidiscip. J..

[101] Bentler P.M., Bonett D.G. (1980). Significance tests and goodness of fit in the analysis of covariance structures. Psychol. Bull..

[102] Zhang J., Wu W., Zhao X., Zhang W. (2020). Recommended psychological crisis intervention response to the 2019 novel coronavirus pneumonia outbreak in China: A model of West China Hospital. Precis. Clin. Med..

[103] Shigemura J., Ursano R.J., Morganstein J.C., Kurosawa M., Benedek D.M. (2020). Public responses to the novel 2019 coronavirus (2019-nCoV) in Japan: Mental health consequences and target populations. Psychiatry Clin. Neurosci..

[104] Prati G. (2021). Mental health and its psychosocial predictors during national quarantine in Italy against the coronavirus disease 2019 (COVID-19). Anxiety Stress Coping.

[105] Lin C.-Y. (2020). Social reaction toward the 2019 novel coronavirus (COVID-19). Soc. Health Behav..

[106] Harper C.A., Satchell L.P., Fido D., Latzman R.D. (2021). Functional Fear Predicts Public Health Compliance in the COVID-19 Pandemic. Int. J. Ment. Health Addict..

[107] Wolfe M.T., Patel P.C. (2021). Everybody hurts: Self-employment, financial concerns, mental distress, and well-being during COVID-19. J. Bus. Ventur. Insights.

[108] Pakpour A.H., Griffiths M.D., Lin C.-Y. (2021). Assessing Psychological Response to the COVID-19: The Fear of COVID-19 Scale and the COVID Stress Scales. Int. J. Ment. Health Addict..

[109] Usher K., Jackson D., Durkin J., Gyamfi N., Bhullar N. (2020). Pandemic-related behaviours and psychological outcomes; A rapid literature review to explain COVID-19 behaviours. Int. J. Ment. Health Nurs..

[110] Aguiar-Quintana T., Nguyen H., Araujo-Cabrera Y., Sanabria-Díaz J.M. (2021). Do job insecurity, anxiety and depression caused by the COVID-19 pandemic influence hotel employees’ self-rated task performance? The moderating role of employee resilience. Int. J. Hosp. Manag..

